# N_2_O emission reduction in the biological nitrogen removal process for wastewater with low C/N ratios: mechanisms and strategies

**DOI:** 10.3389/fbioe.2023.1247711

**Published:** 2023-11-29

**Authors:** Yawen Xie, Cancan Jiang, Benhai Kuai, Shengjun Xu, Xuliang Zhuang

**Affiliations:** ^1^ Research Center for Eco-Environmental Sciences, Chinese Academy of Sciences, Beijing, China; ^2^ College of Resources and Environment, University of Chinese Academy of Sciences, Beijing, China; ^3^ Sheyang Lexin Agricultural Development Co., Ltd., Yancheng, China; ^4^ Institute of Tibetan Plateau Research, Chinese Academy of Sciences, Beijing, China

**Keywords:** BNR, carbon and nitrogen ratio (C/N ratio), N_2_O emission, anaerobic ammonium oxidation (anammox), heterotrophic denitrification (HD)

## Abstract

Urban wastewater, as the main influent type of Waste Water Treatment Plants (WWTPs), has the characteristic of low carbon to nitrogen ratio (C/N). In the biological nitrogen removal (BNR) process, insufficient carbon source often affects the nitrogen removal efficiency and leads to more N_2_O emissions. We review recent researches on N_2_O emissions in the BNR process of wastewater with low C/N. The availability of carbon sources affects heterotrophic denitrification (HD) and autotrophic nitrification/denitrification processes, which are the main reasons for N_2_O emissions in BNR. For the sustainable development of BNR in WWTPs, we introduce strategies suitable for reducing N_2_O emissions in the BNR process of low C/N wastewater from two aspects: traditional process innovation and new process development. These strategies mainly include carbon source addition, adjustment of aeration strategy, optimization of oxidation ditch and biofilm facilities, and application of Anammox related processes. In the future, it is still necessary to further deepen this research direction through the normalization of N_2_O emission quantification standards, exploration of N_2_O metabolism mechanisms, assessment of environmental effects of emission reduction strategies, and practical application of new processes.

## 1 Introduction

With the rapid pace of urbanization, urban wastewater (also known as domestic wastewater) has become the primary type of wastewater in sewage treatment, characterized by low water pollution load. Especially in regions with abundant rainfall, like in southern China, rainwater gets mixed with sewage in the pipe network, coupled with the illegal discharge of industrial wastewater, resulting in the prevalent issue of low carbon and high nitrogen in sewage (Hu et al., 2019). In recent decades, the influent of most Waste Water Treatment Plants (WWTPs) has exhibited a low C (chemical oxygen demand; COD)/N (total nitrogen; TN) ratio, indicating an insufficient carbon source (Zhang et al., 2018). The average C/N ratios for major cities such as Shanghai and Beijing are 3.3 and 4.0, respectively (Jin et al., 2014; Hao et al., 2015; Sun et al., 2016). Based on the comprehensive investigation results, urban wastewater typically has a COD concentration of less than 200 mg/L and a C/N ratio of less than 4 (Liang et al., 2015).

Excessive discharge of nitrogen and phosphorus in sewage can lead to eutrophication of the water system and harm the water environment. Biological nitrogen removal (BNR) is a primary method for reducing nitrogen emissions in WWTPs. Denitrification microorganisms in BNR usually compete with phosphorus-accumulating organisms (PAOs) for available carbon sources (Meinhold et al., 1999). Therefore, the denitrification efficiency of low C/N ratio urban wastewater is inhibited, making it a crucial research focus in the field of BNR in recent years. In order to improve the efficiency of BNR, the more robust methods are the improved Anaerobic/Anoxic/Oxic (A^2^/O), multi-stage Anaerobic/Oxic (A/O), Membrane Bio-Reactor (MBR) and Membrane Aeration Bioreactor (MABR) to support new BNR processes, such as partial nitrification (PN), partial denitrification (PD), simultaneous nitrification/denitrification, and anammox (Sun et al., 2010), which could reduce the addition of carbon source. Alternatively, adding carbon sources like brewery wastewater, kitchen waste leachate, and waste sludge can supplement the pre-treated wastewater carbon sources (Bodik et al., 2009; Wang et al., 2021).

However, while improving nitrogen removal efficiency, the potential issue of nitrous oxide (N_2_O) emission during BNR may have been overlooked in previous development processes, contributing to the global greenhouse effect (Bogner et al., 2008). According to the IPCC guidelines, global warming potentials (GWP) of N_2_O is 265 times that of CO_2_ over a 100 years time span (Edenhofer et al., 2014), and even lower emission fluxes can generate a considerable amount of carbon footprint. The direct emissions of N_2_O caused by anaerobic decomposition of organic matter and BNR during sewage treatment contribute to 3% of its total global emissions (Zawartka et al., 2020). Therefore, N_2_O emission reduction is of great significance for the further development of BNR processes.

Urban wastewater with a low C/N ratio is more likely to produce N_2_O than wastewater with a high C/N ratio. For example, in an activated sludge sequencing batch reactor (SBR), under the operating condition of Biochemical Oxygen Demand (BOD_5_)/TN ratio of 2.6, the total N_2_O emission in the denitrification stage is about 270 times higher than that when the BOD_5_/TN ratio is 4.5 (Kishida et al., 2004). For the A^2^/O process, increasing the influent C/N ratio significantly reduced N_2_O production during nitrification and denitrification (Yan et al., 2017). In the MBR, reducing the C/N ratio from 10 to 2 leads to a decrease in the efficiency of the denitrification process to 14.7% of the original, concurrently increasing N_2_O emissions in both gaseous and dissolved phases (Mannina et al., 2018a; Mannina et al., 2018b).

For new BNR methods that can improve the nitrogen removal efficiency of low C/N wastewater, like PN, some scholars found that N_2_O production in these methods may exceed the that of traditional BNR processes (Joss et al., 2009; Desloover et al., 2011; Domingo-Felez et al., 2014). Monitoring gas emissions from actual sized suspended sludge PN reactors, it can be known that N_2_O emissions account for 3.7% of the nitrogen load, while the formation of N_2_O during the anoxic stage accounts for 66% of N_2_O emissions (Mampaey et al., 2016). However, the maximum accumulation of N_2_O in the mainstream PD/A process was only 0.7% of the influent nitrogen, much lower than previously reported for conventional nitrification-denitrification or PN processes (Du et al., 2020).

Wastewater with a low C/N ratio is more likely to produce N_2_O during both traditional and new BNR processes. We aim to summarize the studies of N_2_O emission in BNR process of low C/N wastewater in recent years, clarify the impact and mechanism of the C/N ratio on N_2_O emission in different BNR methods, and provide suggestions for effective emission reduction strategies and future studies.

## 2 Effect of C/N ratio on N_2_O emissions in biological nitrogen removal

In recent years, a multitude of studies have examined N_2_O emissions in the BNR process of wastewater with varying C/N ratios (Table 1). Because each BNR process has different reaction principles and characteristics, we have categorized them into three groups: complete nitrification/denitrification, partial nitrification-denitrification (PN-D), and new BNR processes for discussion, and summarized the patterns of N_2_O emission from wastewater with different C/N ratios in various processes. Experimental evidence that urban wastewater with low C/N may have more N_2_O emission will be provided, laying the foundation for scientifically mitigating N_2_O emissions in BNR processes.

**TABLE 1:** Summary of N_2_O emissions and denitrification efficiency of wastewater with lower and higher C/N ratios in BNR processes.

BNR type	Reactor	Treatment	C/N	HRT	N removal (%)			N_2_O emission	N_2_O-N conversion ratio (%)	Reference
					TN	NH_4_^+^	NO_3_^−^			
Denitrification process	Mixed flow reactors	Synthetic wastewater	4.5 (COD/NO_3_^−^)	NA	95–100	NA	NA	Max about 4 mg/L	0–3.0 (NO_3_^−^)	Hanaki et al. (1992)
			1.5		40–50	NA	NA	Max over 30 mg/L	6.0–12.0 (NO_3_^−^)	
Nitrification/denitrification	Non-biofilm reactor	Domestic wastewater	5	24–48 h	92.3	NA	NA	NA	0.14 (TN _load_)	Park et al. (2000)
			2.6		42.4	NA	NA	NA	4.57 (TN _load_)	
	Biofilm reactor		5		98.8	NA	NA	NA	0.12 (TN _load_)	
			2.6		42.2	NA	NA	NA	3.01 (TN _load_)	
Denitrification process	BNP reactor	Synthetic wastewater	8	NA	NA	NA	>99	NA	0.005–0.5 (TN _removed_)	Chung and Chung (2000)
			2		NA	NA	53.4	NA		
Nitrification/denitrification	SBR	High-strength wastewater	5.0–5.5	4 days	NA	NA	NA	NA	<1 (TN _removed_)	Itokawa et al. (2001)
			2.4–3.5		NA	NA	NA	NA	20–30 (TN _load_)	
Nitrification/denitrification	SBR	Swine wastewater	BOD_5_/TN = 4.5	10 days	95.5	>99.9	NA	NA	1.71 (TN _load_)	Islas-Lima et al. (2004)
			BOD_5_/TN = 2.6		50.5	83.3	NA	NA	17.7 (TN _load_)	
Nitrification/denitrification	Ludzack-Ettinger (LE)	Synthetic wastewater	1	2 days	73.8	NA	NA	NA	NA	Hwang et al. (2006)
PN	SBNR		1		81.2	NA	NA	NA	NA	
Denitrification process	SBR	Synthetic wastewater	7	NA	NA	NA	>90	0.07 mg/gMLVSS/h	1.04 (TN _removed_)	Alinsafi et al. (2008)
			3		NA	NA	>90	0.18 mg/gMLVSS/h	5.07 (TN _removed_)	
PN	PN reactor	Effluent of UASB reactor treating concentrated black water	1.6(COD/NH_4_^+^)	1.3 days	NA	NA	−0.60∼−0.39	NA	1.9 (TN _load_)	de Graaff et al. (2010)
Fluidized media type BNR processes	A/O	Synthetic wastewater	3	24 h	NA	NA	NA	50∼100 ppm maximum	NA	Kim et al. (2011)
			1.5		NA	NA	NA	800 ppm maximum	NA	
Aerobic granular sludge	SBR	Synthetic wastewater	9.1	8 h	36	31.2	NA	NA	2.9 (TN _load_)	Quan et al. (2012)
			4.5		15	56.5	NA	NA	6.1 (TN _load_)	
Nitrification/denitrification	A/O SBR	Synthetic wastewater	6.5	NA	71	99	NA	24.5 mg	5.3 (TN _load_)	Hu et al. (2013a)
Nitrification/denitrification	A/O SBR	Synthetic wastewater	14.5	16 h	67.2	93.3	NA	2.3 mg	1.3 (TN _removed_)	Hu et al. (2013b)
			7.5		61.3	96.7	NA	9.6 mg	6.0 (TN _removed_)	
			1.5		18.8	76.5	NA	0.5 mg	1.0 (TN _removed_)	
Nitrification/denitrification	SBR	Synthetic wastewater	4	NA	NA	NA	NA	5.78 mg/L*min	NA	Zhao et al. (2013)
			0		NA	NA	NA	10.65	NA	
Denitrification process	Fluidized bed	Synthetic municipal wastewater	5	0.6 h	95.48	NA	95.67	2.03–3.83 mg/min g VSS	0.53–0.95 (TN _load_)	Eldyasti et al. (2014)
			3.5		82.16	NA	84	7.11 mg/min g VSS	1.57 (TN _load_)	
Separated nitrification and denitrification	SBR	Synthetic wastewater	4	16 h	NA	NA	NA	2.17 mg/g·h	11.98 (NO_2_^−^)	Wu et al. (2014)
			1		NA	NA	NA	1.71 mg/g·h	26.63 (NO_2_^−^)	
Nitrification/denitrification	A^2^/O	Real municipal wastewater	10.3	12 h	89.9	99.5	NA	2.8 mg/day	0.061 (TN _removed_)	Ren et al. (2015)
			3.7		58.1	74.4	NA	504.6 mg/day	6.15 (TN _removed_)	
Denitrification process	Biofilter (plexiglass)	Synthetic wastewater	3	NA	92.2	NA	NA	0.2 g m^−3^ h^−1^ maximum	NA	Zhang Y et al. (2016)
			1		NA	NA	57.9	8.0 g m^−3^ h^−1^ maximum	NA	
			0.65		18.5	NA	42.9	3∼4 g m^−3^ h^−1^ maximum	NA	
Nitrification/denitrification	A/O/A SBBR	Synthetic wastewater	4	17 h	98.3	NA	NA	3.61 mg/L	7.28 (TN _removed_)	Ge et al. (2018)
			2		91.75	NA	NA	16.71 mg/L	34.13 (TN _removed_)	
Nitrification/denitrification	A^2^/O	Real municipal wastewater	7.5	12.7 h	61.56	NA	NA	0.032 mg/L	0.05 (TN _removed_)	Yan et al. (2017)
			3.4		21.69	NA	NA	0.64 mg/L	2.23 (TN _removed_)	
Nitrification/denitrification	IFAS-MBR	Municipal wastewater mixed with synthetic wastewater	10	20.6 h	69.4	90.9	52	NA	0.2 (TN _load_)	Mannina et al. (2018b)
			2		44.2	78.6	14.7	NA	1.16 (TN _load_)	
Denitrification process	Batch tests	Synthetic wastewater	12.85	NA	NA	NA	100	1778 μg N g-VSS^−1^ d^−1^	0 (NO_3_^−^)	Lee et al. (2019)
			2.57		NA	NA	53.1	1,488 μg N g-VSS^−1^ d^−1^	53.1 (NO_3_^−^)	
Nitrification/denitrification	MBR	Municipal wastewater mixed with synthetic wastewater	10	20.6 h	69	81	NA	NA	NA	Mannina et al. (2018c)
			5		≈40	95	32	NA	3.5 (TN _load_)	
CANDO	SBR	Municipal wastewater	5	NA	NA	NA	NA	584	60.8 (NO_2_^−^)	Weissbach et al. (2018)
			3		59.7	NA	NA	600 mgN_2_O-N	62.5 (NO_2_^−^)	
Nitrite denitrification	SBR	Synthetic wastewater	6	21 h	NA	NA	NA	1.976 mg/L	NA	Wang et al. (2019)
			1		NA	NA	NA	9.028	NA	
Anammox	AM (anoxic-MBBR)- A^2^/O	Real municipal wastewater	1.2–7.9	10 h	52.6	NA	NA	NA	0.02–0.08 (TN _removed_)	Li et al. (2019)
CANON	SBBR	Synthetic wastewater	1	48 h	84.1	NA	NA	190.4 μgN_2_O-N·g^−1^ VSS	1.32 (TN _load_)	Yan et al. (2019), Fang et al. (2020)
			0		81.4	NA	NA	228.04 μgN_2_O-N·g^−1^ VSS	1.62 (TN _load_)	
INPDA	Microaerobic-SBBR	Synthetic wastewater	2.5	NA	94.1	98.8	NA	1.11 mg/L	2.22 (TN _load_)	Zhou et al. (2020)
Nitrification/denitrification	A/O SBR	Synthetic wastewater	6.5	NA	NA	100	NA	0.11 mg N	0.32 (TN _removed_)	Yang et al. (2021)
			3.3		NA	57.89	NA	0.31 mg N	88.57 (TN _removed_)	
			1.3		NA	34.31	NA	1.12 mg N	16.97 (TN _removed_)	
PN-D	AN, ON1-ON4	Landfill leachate	3.45	4.8 days	78	NA	NA	NA	2.4 (TN _load_)	Gao et al. (2022)

NA, not available.

### 2.1 Complete nitrification/denitrification

Researchers began to investigate the impact of the C/N ratio on N_2_O production during BNR as early as around 2000. At first, the influent of urban wastewater was simulated at a laboratory scale to measure N_2_O. During the denitrification process of the mixed flow reactor, when the COD/NO_3_^−^-N ratio was 1.5 or 2.5, 3%–12% of the influent NO_3_^−^-N was converted into N_2_O. When the COD/NO_3_^−^-N ratio was 3.5 or 4.5, however, the N_2_O conversion rate dropped below 4% (Table 1). Additionally, an insufficient sludge retention time (SRT) was also likely to enhance N_2_O emissions (Hanaki et al., 1992). Studies of cyclic operation demonstrate that for intermittent aeration wastewater treatment systems, the maximum N_2_O emission rate occurs during the initial aerobic stage, not the anaerobic stage (Park et al., 2000).

For the intermittent aeration BNR process of high concentration wastewater, in a bioreactor with an influent COD/N ratio less than 3.5, 20%–30% of the influent nitrogen is discharged in the form of N_2_O; However, with the increase of the C/N ratio to 5.0–5.5, the N_2_O conversion rate dropped below 1% (Itokawa et al., 2001). The nitrification/denitrification process of aquaculture wastewater also has a similar trend. When the C/N ratio of wastewater increased from 2.6 to 4.5, the conversion rate of N_2_O to influent nitrogen decreased from 17.7% to 1.71% (Kishida et al., 2004).

With the advancements of BNR process, the hydraulic retention time (HRT) for nitrification and denitrification has progressively decreased from a maximum of 10 days to less than 1 day. In the complete nitrification process carried by the A/O SBR reactor, when the influent C/N increased from 7.5 to 14.5, the removal rates of TN and NH_4_^+^slightly increased, but the conversion rate of N_2_O (N_2_O-N/TN _removed_) decreased from 6.0% to 1.3% (Hu et al., 2013b). Researchers have also explored a broader range of C/N ratios. When the C/N ratios of synthetic wastewater were 6.5, 3.3, and 1.3, respectively, N_2_O accounts for 0.32%, 88.57%, and 16.97% of the nitrogen loss (TN _loss_) respectively (Yang et al., 2021), indicating that N_2_O emissions do not increase monotonicity as the C/N ratio of influent water decreases, and further in-depth mechanism analysis may be needed to explain the tendency of wastewater with low C/N ratios to have higher N_2_O or overall GHG emissions. For the nitrification and denitrification separation system, the ratio of N_2_O production rate to NO_2_^−^ accumulation rate is defined as the N_2_O conversion rate (rN_2_O-N/rNO_2_^−^-N). When the C/N ratio was 4 and 1, the N_2_O conversion rate was 11.98% and 22.63%, respectively (Wu et al., 2014). The N_2_O emission factor during the nitrification stage ranged from 0.24% to 0.78%, while that of the denitrification stage decreased as the C/N ratio increased, spanning from 12.0% to 26.6%. According to the research findings of other independent nitrification and denitrification processes (Hanaki et al., 1992; Alinsafi et al., 2008; Lee et al., 2019), it can also be observed that the N_2_O emissions from the denitrification process are generally greater than those from the nitrification process. Different studies have different nitrogen indicators compared to the generated N_2_O when defining the N_2_O conversion rate for different processes. Generally, influent nitrogen content (TN _load_) and nitrogen removal (TN _removed_ or TN _loss_) are taken as reference. Considering that most studies employ different statistical units for N_2_O emissions and emission rates, the N_2_O conversion rate can serve as a relatively unified quantitative standard for N_2_O emissions in BNR processes. However, when comparing emissions across different studies, it is necessary to take into account the variations in the conversion rate based on nitrogen indicators for scientifically accurate comparisons.

In addition, monitoring the dynamic changes in N_2_O emission concentration at different stages of the nitrogen removal process and paying attention to emission peaks can provide insights into the emission situation in wastewater treatment with different C/N ratios. In the process of nitrite denitrification, when COD/N was 6, the cumulative amount and duration of N_2_O were significantly lower than that when COD/N is 1 or 4. The peak N_2_O concentrations at COD/N ratios of 6 and 1 were 1.976 and 9.028 mg/L, respectively (Wang et al., 2019). These values can serve as a basis for characterizing emission characteristics.

**FIGURE 1:**
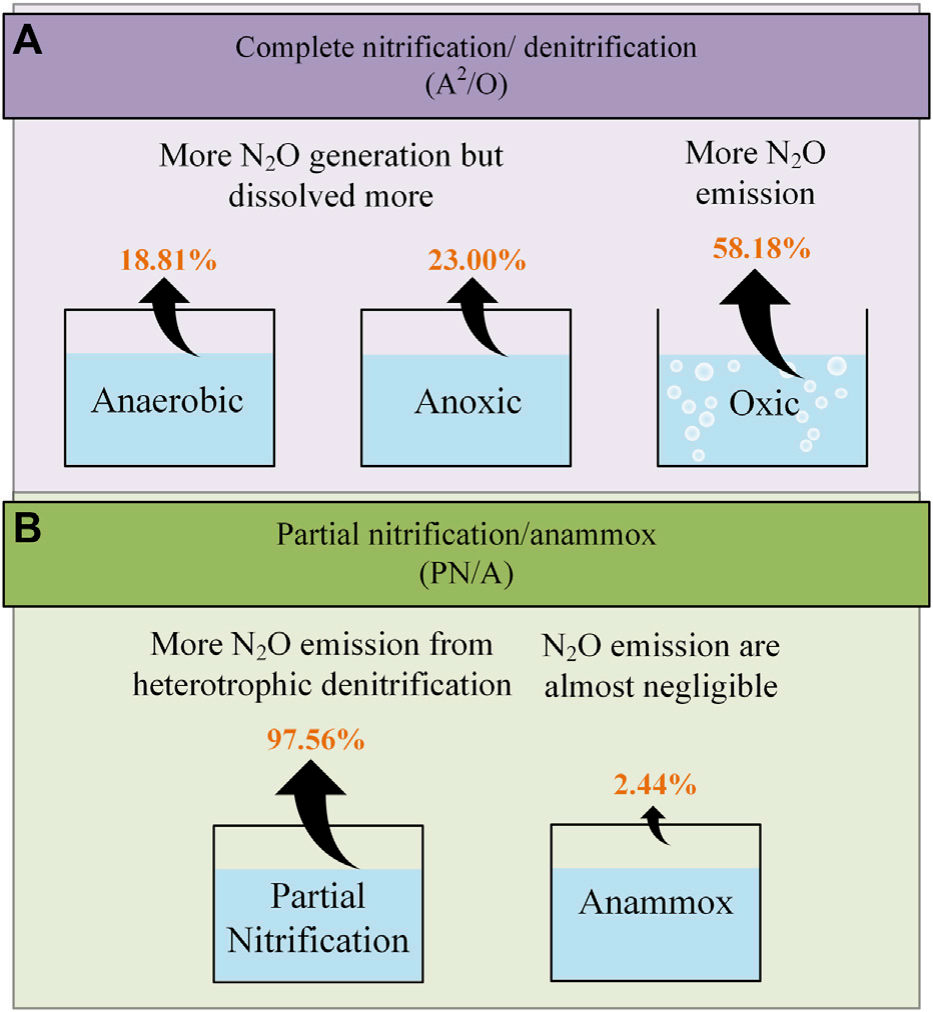
N_2_O generation and emission in different processes. **(A)** Complete nitrification/denitrification (A^2^/O); **(B)** Partial nitrification/anammox (PN/A). The proportions of N_2_O emissions from each reactor depicted in the figure are average values based on measured values found in the relevant literature (refer to Tables 1–3). It is important to note that these estimates are provided for reference purposes only.

Recently, N_2_O emissions during BNR of actual wastewater with different C/N ratios have also been monitored. Continuous monitoring of N_2_O emissions from SBR reactors in sewage treatment plants for 1 year revealed a significant positive correlation between N_2_O emissions and influent COD/N (*R* ^2^ = 0.346, *p* = 0.044 < 0.05), except for August and September. A lower influent COD/N (less than 6) corresponded to a higher N_2_O emission (Sun et al., 2013). Similarly, in an A^2^/O bioreactor system, when the influent C/N decreased from 10.3/10.7 to 3.5/3.8, N_2_O-N conversion rate increased from 0.043%–0.061% to 6.15%–9.18% (Ren et al., 2015). The research on A^2^/O system in 2017 also found that N_2_O emission and generation and the total conversion rate of N_2_O-N decreased significantly with the increase of influent C/N. And N_2_O was mainly produced through the denitrification process in anaerobic and anoxic ponds (Yan et al., 2017) (Figure 1A).

### 2.2 Partial nitrification/denitrification

In order to improve nitrogen removal efficiency and reduce resource input, partial nitrification/denitrification (PN-D) process (or shortcut biological nitrogen removal-denitrification, SBNR-D) came into being. The BNR process involves partially oxidizing ammonia to nitrite and directly reducing nitrite to nitrogen (N_2_) without requiring complete oxidation through NO_3_^−^-N (Schmidt et al., 2003). PN reduces the oxygen required by 25%, and the carbon source required for denitrification by 40% (Tseng et al., 1998; Pollice et al., 2002), making it an economical alternative to the complete nitrification/denitrification process. Due to the low dissolved oxygen (DO) concentration and high NO_2_^−^ concentration increasing N_2_O emissions in the PN process, which may attribute to heterotrophic denitrification. N_2_O emission characteristics may differ from those of the complete nitrification process (Gabarro et al., 2014).

In 2006, researchers measured N_2_O emissions from laboratory scale SBNR processes. For wastewater with a C/N ratio of 1, the process was able to remove 81.2% of TN. And its N_2_O is mainly produced in the anoxic denitrification section, but the emissions were over 90% lower than that of the complete nitrification process under the same conditions (Hwang et al., 2006). For the UASB reactor with a COD/NH_4_^+^ ratio of 1.6 treating the effluent of concentrated black water, PN exhibits a similar effect, with the N_2_O conversion rate accounting for 1.9% of the TN _load_ (de Graaff et al., 2010). In the continuous aeration PN process, for the wastewater with a C/N ratio of 6, the N_2_O emissions were found to range from 18.67 to 330.09 μg/h, and the conversion rate (N_2_O-N/NH_4_^+^-N) was relatively low (0.42%) (Liu et al., 2021a), which also reflected the advantages of PN-D in reducing N_2_O emissions during wastewater treatment.

### 2.3 New BNR process

In recent years, in addition to complete nitrification/denitrification and PN-D to improve efficiency, several new BNR processes have been developed, such as Completely Autotrophic Nitrogen removal Over Nitrite (CANON) process, Coupled Aerobic-anoxic Nitrous Decomposition Operation (CANDO), Anammox related process, n-DAMO (Nitrate-dependent anaerobic methane oxidation) process, etc. These new processes minimize the demand for organic carbon and the oxygen required for nitrification (Horstmeyer et al., 2017), making them more suitable for treating wastewater with low C/N ratios.

In the BNR process dominated by anammox, the enrichment of anammox bacteria by anaerobic carrier biofilm enhanced the denitrification efficiency under C/N = 2.7–5 (Li et al., 2019). However, A^2^/O process combined with denitrification and anammox increased the denitrification efficiency of actual wastewater with low COD/N by about 16.9%. With enhanced denitrification, N_2_O emissions remained relatively low. In the A^2^/O process combined with anoxic carrier biofilm (AM-A^2^/O), the actual N_2_O conversion rate for wastewater with C/N ratios ranging from 1.2 to 7.9 was only 0.02%–0.08% (Li et al., 2019). The maximum N_2_O emission during sewage treatment with low COD/N (2.7 ± 0.4) was only 0.15 mg/L (Li J W et al., 2020). Recently, researchers have combined nitrification, PN and anammox into a comprehensive process, called INPDA (integrated nitrification, partial denitrification and anammox). The TN removal rate of this process could reach 94.1%, and the N_2_O conversion rate (N_2_O-N/TN _load_) for treating wastewater with a C/N of 2.5 is calculated to be 2.2% (Zhou et al., 2020). Compared with previous processes in A^2^/O or A/O sequencing batch biofilm reactors (SBBR), the above combined processes have lower N_2_O emissions when treating wastewater with low C/N.

For the CANDO process that focuses on eliminating NO_2_^−^, when the C/N ratio was 3, the N_2_O yield (N_2_O-N/NO_2_^−^-N) was 62.5%, and when the COD/N ratio was 5, the N_2_O yield decreased to 60.8%, accompanied by an increase in the nitrogen removal rate (Weissbach et al., 2018). In the unipolar CANON system, a similar pattern was observed. When the C/N ratio decreased from 1 to 0, the total N_2_O emissions increased from 1.32% to 1.62%. Comparisons reveal that carbon source has no significant impact on N_2_O emissions during hydroxylamine (NH_2_OH) oxidation and heterotrophic denitrification (HD). The enhancement of nitrifying bacteria denitrification in wastewater with lower C/N ratio is the primary reason for the rise in total N_2_O emissions in this process (Fang et al., 2020).

Summarizing the N_2_O emissions from BNR in different processes, it can be seen that the processes of low C/N wastewater treatment tend to result in higher N_2_O production, whereas new processes like anammox combined with PN or PD and treatment systems with added biofilm exhibit lower N_2_O conversion rates under low C/N conditions.

## 3 N_2_O emission mechanism of wastewater with low C/N ratio

Based on the monitoring and research results of emissions, we found that low C/N wastewater tends to emit more N_2_O in BNR compared to high C/N wastewater, greatly enhancing the greenhouse effect of BNR process. Consequently, it is imperative to investigate the factors responsible for this pattern and understand how the decreased carbon load in urban wastewater affects the metabolic pathway of N_2_O.

### 3.1 Heterotrophic denitrification

Microbial-mediated denitrification plays a crucial role in the denitrification of wastewater. Nitrate (NO_3_^−^) and nitrite (NO_2_^−^) are reduced to harmless nitrogen gas (N_2_). This process is usually mediated by heterotrophic bacteria (HB), including four steps mediated by nitrate reductase (NAR), nitrite reductase (NIR), nitric oxide reductase (NOR) and nitrous oxide reductase (N_2_OR) respectively (Zumft, 1997). HB utilize carbon sources as electron donors. Specifically, after decomposing the carbon source in the environment, these denitrification bacteria can store the carbon source in the form of polyhydroxyalkanoates (PHAs) and glycogen in the anaerobic stage, as electron donor for endogenous denitrification, used for biomass production and anaerobic reduction of nitrogen oxides, and generate energy (Jetten et al., 1997; Oehmen et al., 2005; Miao et al., 2015).

**FIGURE 2:**
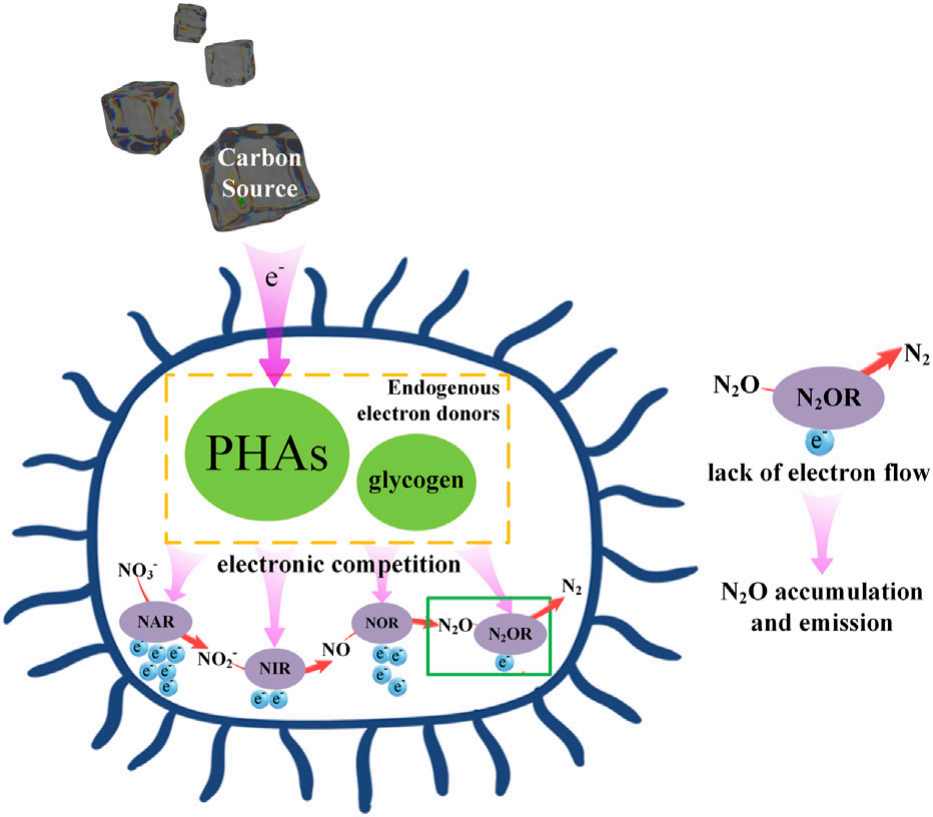
The Mechanism of carbon source regulating nitrogen metabolism in heterotrophic denitrification.

When the denitrification process is not in optimal balance, the intermediate gaseous products nitric oxide (NO) and nitrous oxide (N_2_O) may be discharged into the environment. Among them, N_2_O is generated through the sequential action of NO_3_^−^, NO_2_^−^, and NO reductase (Richardson et al., 2009) (Figure 2). Taking *Alcaligenes faecalis*, a denitrification culture, as an example, N_2_O emissions are higher in the absence of carbon sources; When the electron donor is increased due to the addition of carbon source, the output of N_2_ increases, but the production of N_2_O does not increase (Schalk-Otte et al., 2000), which proves that HB may not be able to complete the denitrification process under the condition of insufficient carbon source, and incomplete denitrification makes a large amount of intermediate N_2_O produced. However, the inclusion of a carbon source facilitates complete denitrification and the generation of the final product, N_2_. It was observed in batch experiments that N_2_O accumulated at C/N ratios of 1.28 and 2.57, while complete denitrification occurred at C/N ratios of 5.14 and 12.85 (Lee et al., 2019).

In the process of denitrification, when the electron supply rate of the oxidation process cannot meet the demand for electrons of the four reduction steps, electronic competition will occur between the four reduction steps (Richardson et al., 2009), so the availability of electrons can adjust the activities of various denitrification enzymes. In research, PHAs are commonly used as the primary endogenous electron donor, serving as a bridge between the external carbon source and the electron utilization of denitrification enzymes. The electronic availability in the system can be known by monitoring the PHAs content, and the PHAs will increase with the increase of influent C/N, which will alleviate the competition of each denitrification process for carbon to some extent (Ge et al., 2018).

Specifically, N_2_OR competes with NOR and NIR for electrons. An additional electron supply can enhance the electron pool of cytochrome C, thereby reducing competitive pressure and boosting N_2_OR activity (Schalk-Otte et al., 2000). Therefore, under different carbon load conditions, it may be the electronic competition intensity between denitrification reductases, rather than the C/N ratio itself, that determines the N_2_O accumulation in the process of denitrification (Lu and Chandran, 2010).

Further experiments have demonstrated that electronic competition occurs not only under carbon-limiting conditions but also in situations with excessive carbon sources. This is because the various denitrification processes are interrelated. In the traditional concept, BNR may be carried out by a variety of organisms in sequence, for example, one denitrification bacterium converts nitrate into nitrite, and then another denitrification bacterium converts nitrite into nitrogen (Zumft, 1997). Is there no electronic competition between nitrate reductase and other reductases in subsequent steps of denitrification?

The current view is limited. The fact is that over 60% of the colonies in the denitrification enrichment belong to Sphingobacteriales and Flavobacteriales, and the bacteria of this order have complete nitrogen removal pathways (Caspi et al., 2012). All denitrification enzymes compete for electrons from a common electronic supply system. As the carbon loading rate changes, electrons will be differentially distributed among various reductases, and when the electron flux of reducing nitrite is greater than that of reducing N_2_O, N_2_O will accumulate (Pan et al., 2013a). In the same year, the research team developed an electron carrier model, indicating that carbon oxidation provides electrons for carriers, and nitrogen oxides receive electrons from these carriers for reduction. The carbon oxidation process and nitrogen reduction process are closely connected. This model enhanced the prediction of N_2_O accumulation ability during denitrification by employing different affinity constants and reduction carriers to describe the relative competitive ability of electrons in each denitrification step (Pan et al., 2013b).

Nitrous oxide reductase (N_2_OR) is the sole enzyme responsible for catalyzing the decomposition of N_2_O into N_2_, and the structural gene nosZ encoding N_2_OR is co-transcribed with nosR. N_2_OR is not only influenced by the electronic competitive activity of other denitrification reductases but is also highly sensitive to DO in the environment. The anoxic and anaerobic enrichment conditions will also lead to N_2_O accumulation (Conthe et al., 2018).

### 3.2 Autotrophic nitrification/denitrification

**FIGURE 3:**
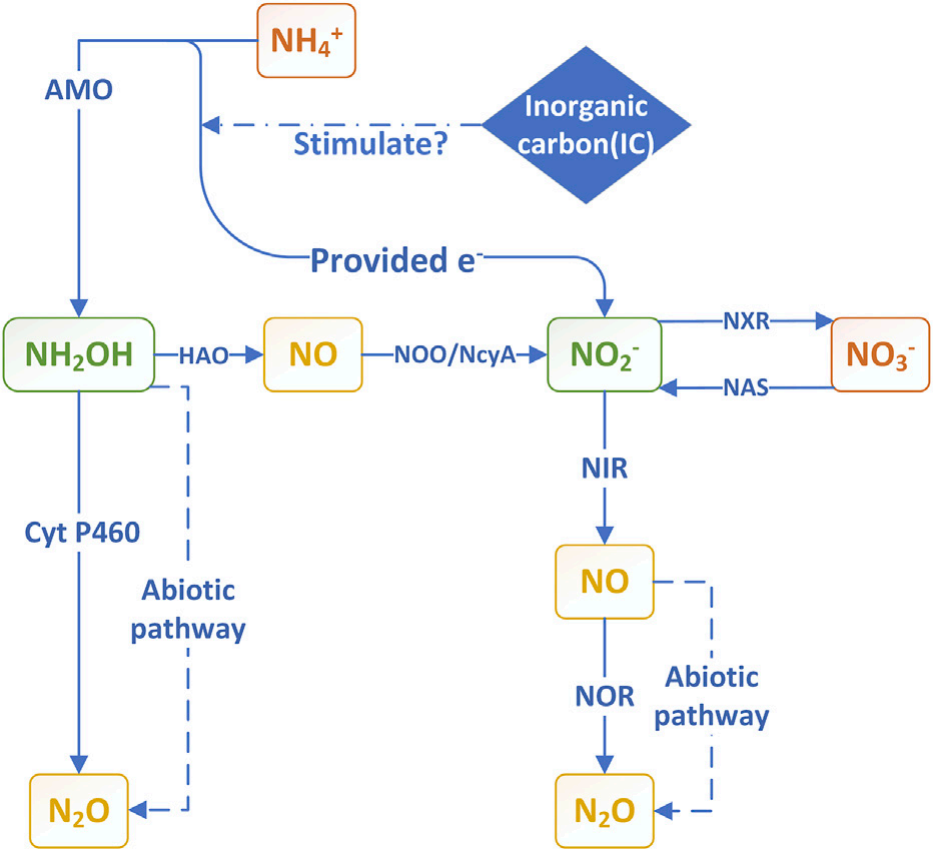
The main pathway of N_2_O emission and the role of carbon sources in autotrophic nitrification/denitrification.

In addition to the contribution of HB, N_2_O emission from BNR processes are partially attributed to ammonia oxidizing bacteria (AOB) and ammonia oxidizing archaea (AOA) during the nitrification process in the mixed system. Under low C/N conditions, it was found that the contributions of heterotrophic denitrification activity and autotrophic nitrification activity to N_2_O production were similar. Therefore, some researchers have created a process model that includes both heterotrophic and autotrophic denitrification paths, resulting in a slightly better prediction effect for N_2_O emissions compared to the single-path denitrification model (Domingo-Felez et al., 2017). In the process of AOB metabolism, the oxidation of NH_2_OH produces a byproduct N_2_O (Chandran et al., 2011); similarly to HB, it uses ammonia or hydrogen as the electron donor to reduce nitrite, resulting in the production of N_2_O (Figure 3). This autotrophic ammonia oxidizing bacteria with denitrification ability is primarily classified as *Nitrosomonas* (Bock et al., 1995), and N_2_O is the end product of its nitrogen metabolism (Kim et al., 2010; Law et al., 2012). The model shows that in most cases, the AOB denitrification pathway is dominant, while the NH_2_OH oxidation pathway becomes more significant at high DO levels (e.g., 3.5 mg O_2_/L) (Peng et al., 2015a).

Unlike heterotrophic organisms, the nitrogen metabolism pathway of autotrophic AOB bacteria is affected by the availability of inorganic carbon (IC). In nitrifying sludge rich in AOB and nitrite oxidizing bacteria (NOB), autotrophic growth can lead to a lack of IC, which in turn limits the oxidation activity of AOB towards NH_3_ (Todt and Dorsch, 2016). In the PN/A process, N_2_O is also produced through pathways associated with AOB. Decreasing IC/N will inhibit the activity of AOB, thereby enhancing N_2_O generation through NH_2_OH oxidation (Ma et al., 2015; Li L et al., 2020) (Figure 2). Enhancing the availability of IC will result in a higher reaction rate between AOB and related N_2_O (Peng et al., 2015b). However, further research is needed to elucidate the mechanism of N_2_O generation under different IC conditions, and more evidence is required to support the IC/N concentration that can achieve N_2_O emission reduction in actual wastewater treatment.

In the treatment of low C/N wastewater, certain specific conditions can cause an increase in N_2_O production of AOB. If the nitrite concentration in the influent is high, it will promote the denitrification of AOB and effectively convert nitrite to N_2_O (Colliver and Stephenson, 2000). However, the limited COD availability in low C/N wastewater will also cause nitrite accumulation (Hanaki et al., 1992), further enhancing the N_2_O generation pathway of AOB denitrification. Other conditions, such as low pH, can cause nitrification stress in AOB, leading to protonation of NO_2_^−^ into HNO_2_. HNO_2_ inhibits the activity of NOB in the mixed system, causing the accumulation of NO_2_^−^, which in turn promotes N_2_O production (Peng et al., 2015b). Additionally, nitrifying sludge is more likely to produce N_2_O from AOB under anaerobic conditions. Experiments have shown that the N_2_O generation rate reaches its maximum at a DO of 0.85 mg O_2_/L, while the N_2_O emission factor decreased with an increase in DO from 0.35 mg O_2_/L to 3.5 mg O_2_/L (Peng et al., 2015a).

In terms of metabolism and gene expression, the AOB denitrification pathway includes NIR that reduces NO_2_^−^ to NO and NOR that reduces NO to N_2_O (Kozlowski et al., 2016b). Studies on bacteria, such as eutrophic *Nitrosomonas europaea*, have found that AOB requires NOR activity to convert NO into N_2_O during the processes of nitrification and denitrification, that is, it is not the nitrite reductase gene NirK that is necessary for N_2_O production, but the nitric oxide reductase gene NorB (Kozlowski et al., 2014; Kozlowski et al., 2016a). For oligotrophic AOB and AOA lacking NOR activity, it is more important that abiotic reactions (chemical denitrification) convert NO into N_2_O and discharge it *in vitro* (Kozlowski et al., 2016a). Recent experiments have proven that N_2_O in PN with higher nitrogen removal efficiency for low C/N wastewater was also produced by mixing biological and abiotic nitrosation (Terada et al., 2017). During the aerobic ammonia oxidation process, extracellular NH_2_OH undergoes a non-biological reaction with substances in the growth medium, which also serves as a pathway for N_2_O conversion (Liu et al., 2017). Previous studies may have underestimated N_2_O emissions caused by non-biological pathways.

The nitrification of AOB is achieved by the membrane-bound enzyme ammonia monooxygenase (AMO) oxidizing ammonia (NH_3_) to produce NH_2_OH, which is then mediated by the periplasmic enzyme hydroxylamine dehydrogenase (HAO) to produce nitrite. The acidic conditions mentioned earlier (pH < 5) can induce partial inhibition of HAO, and the released NO can be further reduced to N_2_O, thereby enhancing the NH_2_OH oxidation pathway of N_2_O (Jiang and Bakken, 1999).

There remains a research gap regarding the N_2_O emission contribution of AOA in BNR of WWTPs. It is known that both AOA and AOB increase in high ammonium states in soil ecosystems, while AOA dominates in low ammonium states (Hink et al., 2018). The advantages of AOA can be demonstrated under conditions of low ammonium, hypoxia, long SRT, and high temperature, indicating that AOA may promote the generation of N_2_O to a certain extent under these conditions (Wu et al., 2020). However, currently only Castellano-Hinojosa et al. (2018) have reported a negative correlation between AOA abundance and N_2_O emissions in four aerobic sludge wastewater treatment plants in Spain. Therefore, they believe that AOA is unlikely to make a significant contribution to N_2_O generation.

### 3.3 Contribution of various factors to N_2_O emissions

For nitrogen removal systems with integrated functions of autotrophic and heterotrophic microorganisms, denitrification is the main contributor to N_2_O emissions. For example, in the SBBR reactor for treating low C/N (=0∼1) wastewater, N_2_O emissions from hydroxylamine oxidation, AOB denitrification, and heterotrophic denitrification accounted for 5.4∼7.6%, 45.2∼60.8% and 33.8∼47.2% of the total N_2_O emissions, respectively. In reactors with varying C/N ratios, the contribution of denitrification to N_2_O production ranged from 90% to 96%. And with the decreases of the C/N ratios, the total amount of N_2_O in denitrification process increased (Domingo-Felez et al., 2017; Yan et al., 2019; Fang et al., 2020). However, NH_2_OH oxidation and AOB denitrification pathways are primarily found in the PN process (Ni and Yuan, 2015). In the aerobic stage, N_2_O generated by NH_2_OH oxidation accounts for 65% of the total N_2_O, and the N_2_O generated by AOB denitrification in the later stage is nearly identical to that generated by NH_2_OH oxidation (Rathnayake et al., 2013). In addition, N_2_O emissions from non-biological pathways account for 1.1% of the TN _load_ (Soler-Jofra et al., 2016; Liu et al., 2018).

In the partial nitrification-anammox (PN/A) process, which enhances denitrification performance, anammox process also emits a small amount of N_2_O in addition to the contribution of PN. The most probable N_2_O emission path involves heterotrophic denitrification in anammox particles (Okabe et al., 2011). The research showed that in the primary PN/A particle reactor, 70% of N_2_O emissions occurred in the aerobic surface area dominated by AOB, and 30% occurred in the anoxic area dominated by anammox, and NH_2_OH oxidation and AOB denitrification had a similar proportion of contributions to N_2_O emissions from AOB related pathways (Ali et al., 2016). In the full-size two-stage PN/A reactor, N_2_O emissions in PN stage were high (1.2% of TN _load_), and the emission source could be located in HD (Hausherr et al., 2022). Due to the fact that the majority of N_2_O (approximately 97.5%) was emitted by PN units, the emissions of N_2_O from anammox could be almost negligible (Desloover et al., 2011; Okabe et al., 2011) (Figure 1B). In addition, for the monopolar anaerobic nitrogen removal process dominated by Anammox, the batch experiment of ^15^N isotope tracing and specific inhibitors demonstrated that when nitrite was the main nitrogen source, the N_2_O emissions of HD and AOB denitrification were 64% and 36%, respectively (Li et al., 2017).

Under aerobic conditions, little research is available on enzyme-based N_2_O metabolism. The primary metabolic pathway of N_2_O is HD, with the majority of escaping N_2_O originating from AOB. This indicates that the rate of N_2_O consumption by HB is lower than that of N_2_O production by AOB, which is the main reason for N_2_O emission from aerobic phase (Yang et al., 2021). In SBR with intermittent aeration, it is also found that AOB denitrification is the main way to produce N_2_O, and HD is the sink of N_2_O (Liu et al., 2021a).

Due to the unclear correlation between microbial community abundance and N_2_O generation contribution, it is challenging to make a unified and clear judgment on the N_2_O generation mechanism under different denitrification processes for urban wastewater with low C/N ratios. Future research needs to start from multiple factors such as microbial community structure, N_2_O metabolic characteristics, enzyme activity, and more in-depth consideration of various N_2_O production pathways and their representative microbial contributions, in order to lay a solid scientific foundation for proposing N_2_O emission reduction strategies for low C/N wastewater treatment. In terms of methodological applications, emerging single cell metabolic phenotypes can be considered, whose single cell Raman spectroscopy (SCRS) can identify and select individual cells or functional bacterial populations (Jones et al., 2019; Liu et al., 2022), enabling *in situ* functional analysis. The key relationship between phenotypic heterogeneity and plasticity of PAO populations in enhanced biological phosphorus removal (EBPR) systems and the stability of the EBPR process has been investigated using SCRS, which cannot be solely determined through phylogenetic analysis (Li et al., 2018). Practical experience has shown that SCRS can be widely applied in the fields of biology and environment, addressing key limitations related to omics-centered environmental ecological research methods, such as a lack of cell-level resolution and limited capacity to infer gene functional relationships, especially for highly diverse and featureless ecosystems (Wang et al., 2020). If relevant cutting-edge technologies are employed to directly correspond different bacterial communities and their N_2_O production and reduction functions, it could provide a better understanding of the N_2_O metabolism mechanism in BNR systems.

## 4 N_2_O emission reduction strategy of traditional biological nitrogen removal process

The traditional BNR process, represented by complete nitrification/denitrification and PN-D, has been widely used in WWTPs. For the treatment of urban wastewater with low C/N, most efforts still focus on upgrading and renovating existing WWTPs. Therefore, it is particularly important to optimize emission reduction measures based on the N_2_O emission characteristics of low C/N wastewater for practical applications.

### 4.1 Carbon source dosing

For the denitrification process in SBR reactors, the N_2_O production characteristics of microbial systems established on different carbon sources vary. Lack of oxygen inhibits the production of NAR in the methanol carbon source system, leading to reduced N_2_O production in the anoxic and subsequent aerobic stages. However, in a carbon source system where ethanol is used, NAR is more resistant to oxygen limitations. The downstream N_2_OR is more sensitive to hypoxia and inhibition than other denitrification enzymes (Korner and Zumft, 1989), which makes N_2_O generated and accumulated in the subsequent aerobic stage. The above differences may be related to the distinct composition of microbial communities established by methanol and ethanol. Therefore, in the practical operation of WWTPs, strict control of ethanol addition to the anoxic zone is required to minimize the production and discharge of N_2_O in the downstream aerobic zone. For the modified Ludzak Ettinger (MLE) processes dominated by denitrification, changing the carbon source from methanol to acetate can reduce the N_2_O conversion rate from 3.0% to 1.0%, and the N_2_O reduction rate of acetate biomass is higher than that of methanol biomass (Table 2). The disparity in N_2_O reduction rate could be attributed to different bacterial communities enriched with different carbon sources (Song et al., 2015). Then some scholars used methanol, sodium acetate and glucose as external carbon sources to optimize C/N of denitrification biofilter. Through comprehensive comparison, it is also confirmed that sodium acetate is more suitable as an external carbon source (Xu et al., 2018).

**TABLE 2:** Optimization strategies for N_2_O emission reduction operation of various BNR processes for low C/N wastewater treatment.

BNR type	Reactor	C/N	Emission reduction strategies	N_2_O-N conversion ratio (%)		N removal (%)	Reference
				Original	After reduction		
Nitrification/denitrification	A/O SBR	1.5–4.0 (COD/NH_4_^+^)	Add carbon source appropriately	1.0 (TN _removed_)	0.5 (TN _removed_)	35.6	Hu et al. (2013b)
		7.5–14.5		6.0 (TN _removed_)	1.3 (TN _removed_)	67.2	
Denitrifying fluidized bed bioreactors (DFBBRs)	DFBBR	5	Increase biofilm thickness	0.95 (TN _load_)	0.53 (TN _load_)	96	Eldyasti et al. (2014)
Cyclic Activated Sludge System (CASS)	CASS reactors	4.2	Continuous feeding instead of batch feeding	28.2 (TN _load_)	16.3 (TN _load_)	45.8–53.7	Liang et al. (2015)
			The aeration rate increased from 20 L/h to 50 L/h	16.3 (TN _load_)	9.1 (TN _load_)	45.8	
Modified Ludzak Ettinger (MLE) processes	A/O	NA	Replacing methanol with acetate as a carbon source	2.3 (TN _load_)	1.3 (TN _load_)	90.8	Song et al. (2015)
PN/A	Full-scale granular sludge reactor	NA	Continuous aeration instead of intermittent aeration	2.5 (TN _load_)	1.0 (TN _load_)	74.4	Castro-Barros et al. (2015)
Nitrification/denitrification	A^2^/O	4.4	The internal recycle ratio decreased from 300% to 100%	0.21 (TN _load_)	0.14 (TN _load_)	45.8	Yan et al. (2016)
Aerobic nitrifying granular sludge	SBR	5/3 (COD/NH_4_^+^)	Under the temperature of 22.3°C, pH of 7.1 and aeration rate of 0.20 m^3^/h	0.5(NH_4_^+^)	less than 0.01(NH_4_^+^)	50.0	Liu et al. (2016)
Simultaneous nitrogen and phosphorus removal	A/O/A SBBR	1.0∼4.0	Add carbon source appropriately to C/N = 4	26–35 (TN _removed_)	7.28 (TN _removed_)	98.3	Ge et al. (2018)
CANDO	An automated bioreactor system	3.0–5.0	Add carbon source appropriately to C/N = 5	65.7 (NO_2_^−^)	60.8 (NO_2_^−^)	59.7	Weissbach et al. (2018)
CANON	SBBR	0–1	Add carbon source appropriately to C/N = 1	1.62 (TN _load_)	1.32 (TN _load_)	84.1	Yan et al. (2019)
Nitrification/denitrification	SBBR	2.76	Carbon source (methanol) step-by-step dosing instead of one-time dosing	6.26 (TN _load_)	3.4 (TN _load_)	83.3	Chai et al. (2019)
PN-D	SBR	6	Intermittently aerated mode instead of continuously aerated mode	0.42 (TN _load_)	0.19 (TN _load_)	93.5	Liu et al. (2021a)
Nitrification/denitrification	Continuous flow experiments	3	Have a microbial weak electrical stimulation of 0.2 V	2.2 ppm	0.8 ppm	Increase 20%	Dong et al. (2022)
Nitrification/denitrification	A + OD	2.57	Use the pre-anaerobic carrousel oxidation ditch (A + OD)	NA	0.14 (TN _removed_)	75.5	Ren et al. (2013)
Nitrification/denitrification	Carrousel OD	5(COD/NH_4_^+^)	Use the pilot-scale Carrousel oxidation ditch	NA	0.027 (TN _load_)	59.92	Zheng et al. (2015)
Nitrification/denitrification	OD	5	Use the pilot-scale oxidation ditch	NA	0.142 (TN _load_)	57.75	Zheng et al. (2021)
Nitrification/denitrification	UCT-MBR	5	Configure biofilm	NA	0.5 (TN _load_)	NA	Mannina et al. (2018c)

Compared with one-time dosing, incremental addition of carbon sources enhanced nitrogen removal efficiency and could serve as a strategy for highly automated wastewater treatment systems to reduce N_2_O emissions (Chai et al., 2019). Furthermore, within the cyclic activated sludge system (CASS), consistent feeding can improve denitrification efficiency and decrease N_2_O emissions as compared to intermittent feeding. The carbon source in the continuous feeding alleviates the electronic competition between denitrification reductases in the non-aeration stage (Richardson et al., 2009). From a microbial community perspective, the continuous feeding system exhibits a high abundance of N_2_O reducing bacteria within the denitrification bacteria (Liang et al., 2015). There are significant differences in N_2_O emission reduction strategies among the different processes mentioned above, therefore, when designing emission reduction plans, it is necessary to optimize the design by categorizing the processes.

This indicates that the substitution and addition strategy of additional carbon sources can improve the C/N ratio of wastewater, thereby enhancing denitrification efficiency while reducing N_2_O. It is a straightforward and more cost-effective strategy for reducing emissions in WWTPs.

**FIGURE 4:**
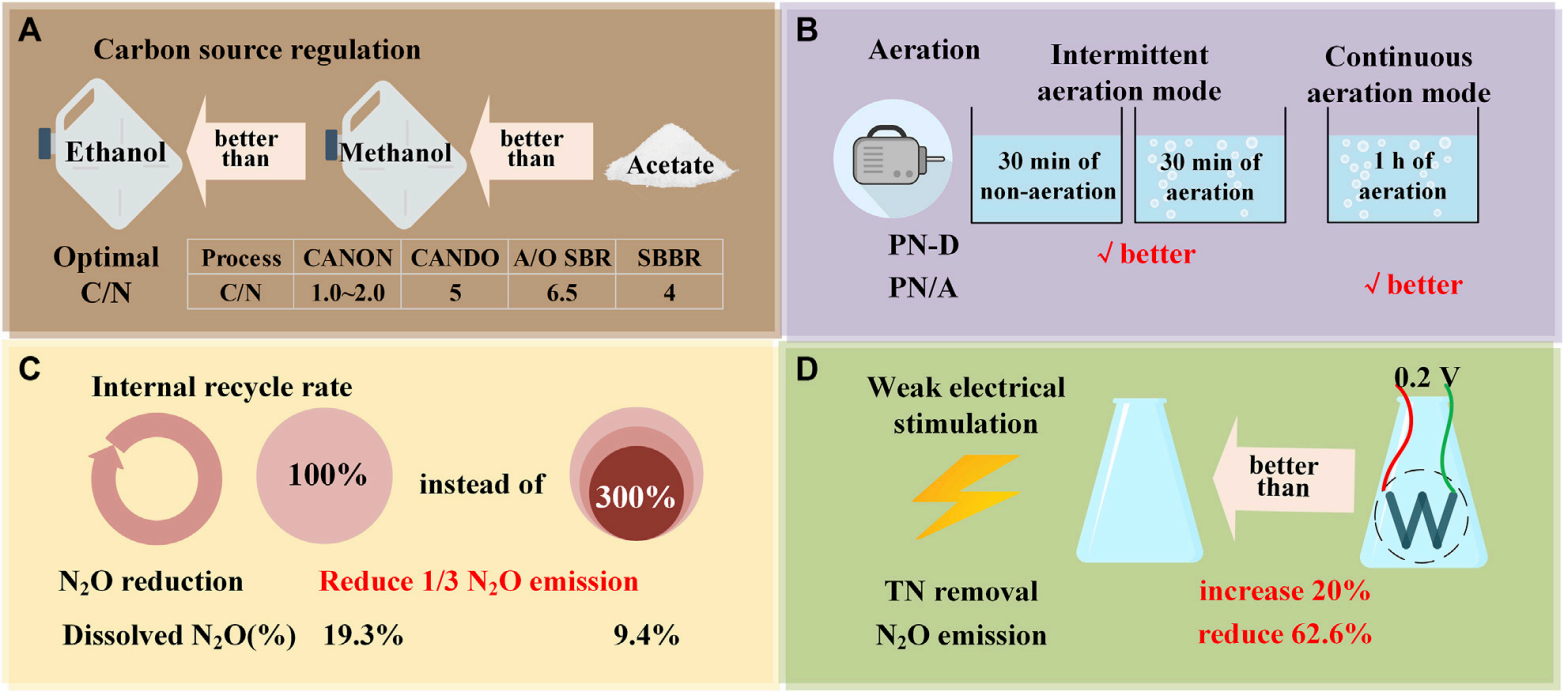
Optimization of process strategies for reducing N_2_O emissions in low C/N wastewater. **(A)** Carbon source regulation; **(B)** Aeration; **(C)** Internal recycle rate; **(D)** Weak electrical stimulation.

In the combined denitrification process of CANON and denitrification, an appropriate carbon source (C/N = 1) could decrease the total N_2_O production by 16.7% compared to C/N = 0. This was due to the inhibition of the AOB denitrification process that consumed NO_2_^−^-N, which accounted for over 94.5% of N_2_O emissions (Yan et al., 2019). Based on the N_2_O emissions of wastewater with low C/N from various processes in Chapter 2, there are significant differences in carbon source dosage and C/N ratio for different processes to achieve emission reduction effects (Figure 4A). For the CANON system, primarily composed of autotrophic microorganisms, adding a small amount of carbon source with C/N ratio of 1 could achieve a significant reduction in N_2_O emission. If a large amount of carbon source (C/N > 1) is added, the heterotrophic NOB activity cannot be inhibited, disrupting the stability of the system (Zhang et al., 2017; Yan et al., 2019). Specifically, due to the predominance of autotrophic denitrification bacteria in this process, the main carbon source required is IC. Considering the weak inhibitory effect of IC/N on AOB activity and the strong inhibitory effect on anammox activity, some studies have shown that using influent with a C/N range of 1.2 to 1.5–2.0 in CANON can achieve stable denitrification effects (Zhang X J et al., 2016; Yue et al., 2018). This indirectly indicates that the CANON process is suitable for treating low C/N wastewater, but the relationship between N_2_O emissions and IC concentration requires further exploration.

In the CANDO that also eliminates NO_2_^−^, when the COD/N ratio increases to 5, the N_2_O yield decreases and the nitrogen removal rate increases compared to C/N = 3 or 4 (Weissbach et al., 2018). Considering the reduction of nitrogen removal efficiency, enzyme activity, and N_2_O emissions, it is more feasible to maintain the influent C/N of A/O SBR at around 6.5 (Yang et al., 2021). Similarly, in another anoxic aerobic BNR experiment, step feeding and additional carbon sources allow AOB (*Nitrosomonas*) to mitigate the denitrification effect of nitrifying bacteria, thereby reducing the N_2_O conversion rate by 66.6% and 12.0%, respectively, indicating that these two methods are effective in reducing N_2_O emissions during sewage treatment (Hu et al., 2013b).

In addition, for the system that simultaneously removes nitrogen and phosphorus, setting the C/N ratio to 4 will result in a minimum N_2_O conversion rate and an ideal nitrogen removal efficiency of 98.3%, but only 27.44% phosphorus can be removed. The phosphorus removal efficiency will reach the optimal value of 82.79% when C/N = 3. This also inspires future research on actual wastewater denitrification to consider the synergistic effects of multiple pollutants removal and N_2_O emission reduction (Ge et al., 2018).

### 4.2 Operating conditions

In the BNR process, the optimization of operating parameters is the key to reducing N_2_O emissions. In the treatment of low C/N (=6) wastewater, compared to continuous aeration, intermittent aeration had a higher TN removal efficiency (93.5% on average), and the N_2_O emission factor decreased from 0.42% of continuous aeration to 0.19%, which promoted PN-D. Among them, the complete ammonia oxidizer (comammox) was significantly enriched during intermittent aeration, and the quantitative results showed that their gene abundance reached 24.7%; The abundance of AOB bacteria significantly decreased (Liu et al., 2021a). Comammox lacks NO reductase, and non-biotransformation emits much less N_2_O than AOB, showcasing its potential for reducing N_2_O emissions (Liu et al., 2017; Kits et al., 2019). Conversely, during the operation of the PN/A process, a transition from low aeration (or hypoxia) to high aeration will quickly increase the N_2_O emission rate, while increased continuous aeration will reduce the emission rate, proving that the continuous aeration strategy is effective in reducing N_2_O emissions in PN/A (Figure 4B) (Castro-Barros et al., 2015).

In the A^2^/O process, for actual domestic wastewater with low C/N (=4.4), when the internal circulation ratio of the process is reduced from 300% to 100%, the production of N_2_O increases from 9.81 × 10^−2^ mg/L reduced to 3.47 × 10^−2^ mg/L (Figure 4C). The primary reduction occurs in the form of N_2_O produced by denitrification in anoxic section. This phenomenon is due to the reduction of the volume of internal circulating liquid and the reduction of nitrate substrate and oxygen that can be used for denitrification. As mentioned in Chapter 4.1, N_2_OR is more sensitive to oxygen, thus, enhancing the activity of N_2_OR entering the anoxic zone, making it more conducive to the denitrification process of reducing N_2_O to N_2_. Experimental evidence indicated that the copy number of nosZ gene increased as the internal circulation ratio decreased from 300% to 100% (Yan et al., 2016).

In addition, electrical stimulation can optimize microbial population structure and enhance microbial autotrophic denitrification (Figure 4D). At the same time, the activity of NAR and NIR is increased to promote denitrification, so as to improve the removal efficiency of NO_3_^−^ and reduce the accumulation of N_2_O. The removal rate of nitrate and TN can be increased by 20%, and the production of intermediate greenhouse gas N_2_O can be reduced by 62.6% when weak electric stimulation (0.2V) is added to the denitrification process of influent C/N = 3 (Dong et al., 2022).

This research suggests that future N_2_O emission reduction strategies can be developed based on N_2_O metabolism mechanisms, such as identifying operating conditions that promote N_2_OR activity or nosZ transcription initiation.

At present, more studies on BNR and N_2_O emission reduction only investigate the effects of individual parameters on emissions, making it difficult to compare the specific effects and overall impacts of various operating conditions. In light of this limitation, researchers have employed Plackett Burman (PB) multi-factor experimental design and response surface methodology (RSM) to explore emission reduction strategies for N_2_O in nitrifying granular sludge systems. The analysis revealed that at a temperature of 22.3°C, the pH value of 7.1, and the aeration rate of 0.20 m^3^/h, the N_2_O emission during the denitrification process is minimal. The predicted results were confirmed using wastewater with COD/NH_4_^+^ = 5/3 (Liu et al., 2016).

### 4.3 Facility enhancement

#### 4.3.1 Oxidation ditch system

In the complete nitrification/denitrification process, the N_2_O emission reduction effect in the BNR process related to oxidation ditch is very significant. In Carrousel oxidation ditch and related pre-anaerobic processes, for influent with C/N ratios of 2.57–5, N_2_O emissions were as low as 0.027%–0.14% of influent nitrogen (Table 3), which was significantly lower than in other complete nitrification and denitrification processes (Ren et al., 2013; Zheng et al., 2015), of which approximately 90% is attributed to nitrification and denitrification. And research on the impact of COD/N on its N_2_O emission characteristics showed that at lower COD/N ratio of 5, the N_2_O emission factor could reach a maximum value of 0.142%, which was higher than the 0.055% at a COD/N ratio of 7 (Zheng et al., 2021). Even though low C/N inflow can promote N_2_O emissions, the oxidation ditch system still plays a significant role in reducing emissions.

**TABLE 3:** New system/process to reduce N_2_O emissions in BNR of low C/N wastewater.

BNR type	Reactor	C/N	N_2_O-N conversion ratio (%)	N removal (%)	Reference
Partial Anammox	MBBR-A^2^/O	1.2∼7.9	<0.08 (TN _removed_)	>73.7	Li et al. (2019)
Two stage N/A	Full-scale nitrification and anammox reactors	NA (municipal wastewater)	2.3 (TN _load_)	NA	Kampschreur et al. (2008)
Two stage PN/A	Up-flow biofilm PN reactor; up-flow granular-sludge anammox reactor	NA (low COD/N ratio)	4.1 (TN _load_)	NA	Okabe et al. (2011)
One-stage PNA	Granular sludge reactor	0.35(COD/NH_4_^+^)	2.0 (TN _load_)	74.4	Castro-Barros et al. (2015)
One-stage PNA	Bio-film MBBR	0.7(COD/NH_4_^+^)	0.35–1.33 (TN _load_)	81	Yang et al. (2016)
One Stage PN/A	SBR	NA	0.98 (TN _load_)	80	Ali et al. (2016)
PD/A	PDA-SBR	0(COD/NO_3_^−^)	0.7 (TN _load_)	71.5	Du et al. (2020)
PN/AM	MBfR	NA	0.34 (TN _load_)	98	Liu et al. (2019)
PND	An anoxic reactor (AN), four aerobic reactors (ON1-ON4), and a settler	3.45	2.4 (TN _load_)	78	Gao et al. (2022)
Denitrifying biofilm/flocs system	Lab-scale biofilm-based reactors	Carbon-Limiting Condition	Decrease 32% N_2_O accumulation	NA	Liu X et al. (2023)
Nitrification/denitrification with strain YR02	SBR	5	Mitigated 98.7% of N_2_O emission	Improved 32% NRE	Wang et al. (2023)
Feammox	Multistage Feammox Bioreactor (MSFB)	2.5	NA	99	Nguyen et al. (2023)

The primary reason why this process can reduce N_2_O emissions is that it can enrich the denitrification bacteria and NOB with a high abundance, which have lower N_2_O emissions (Zheng et al., 2015). However, further evaluation is necessary to determine the emission reduction potential of the process based on the actual operating mode. When the normal operation of the oxidation ditch system is impacted by ammonia overload or aeration failure, the production of N_2_O could significantly increase. Further optimization of operating conditions could also impact N_2_O emissions from the pilot oxidation ditch. The study found that properly extending the SRT to 25 days or immobilizing the aerobic denitrification bacteria PCN-1 on the polyurethane biological carrier to biologically strengthen the oxidation ditch can enrich more comammox belonging to *Nitrospira* in the system, effectively avoid the accumulation of NO_2_^−^, and the system will also express more abundant N_2_O reductase to achieve N_2_O emission reduction (Zhou et al., 2019; Tian et al., 2021).

#### 4.3.2 Biofilm system

Biofilm systems can improve the efficiency of BNR, thereby reducing N_2_O emissions. For example, in a pilot-scale UCT (University of Cape Town) MBR reactor, when treating wastewater with a C/N ratio of 5, the average N_2_O discharge amounts to 0.5% of the influent nitrogen. The enhanced nitrogen removal efficiency of biofilm may be attributed to the coexistence of suspended and attached biomass, as well as the increased richness and diversity of biological communities, which enhance the nitrification and denitrification performance (Eldyasti et al., 2010; Mannina et al., 2018a; Sun et al., 2019). N_2_O can be produced during the oxidation of hydroxylamine and the reduction of nitrite. Due to the mediation and strong influence of microorganisms diffusing within and outside the biofilm on the process area, the thickness of the biofilm has a certain contribution to N_2_O emissions. In the denitrification fluidized bed bioreactor (DFBBR), at a COD/N ratio of 5, the N_2_O conversion rate of the DFBBR system with a biofilm thickness of 680 μm was 0.53% of the total influent nitrogen load, and when the biofilm thickness was 230 μm, the N_2_O conversion rate increased to 0.95%. The sevenfold increase in the concentration of liquid nitrite indicated that the increase in emissions was due to the limited reduction rate of NO_3_^−^ to NO_2_^−^ in thinner biofilms. Therefore, increasing the thickness of the biofilm can reduce N_2_O emissions in granular biofilm processes (Eldyasti et al., 2014).

The biofilm system also promotes the formation of flocs within the system. The flocs of the denitrifying biofilm/flocs system can effectively reduce the total N_2_O accumulation by 32%. The flocs also promote a high proportion of electron distribution to N_2_OR, indicating that the flocs have strong N_2_O reduction ability (Liu Y R et al., 2023).

In addition, incorporating anoxic carrier biofilm in to actual WWTPs can facilitate *in-situ* enrichment of anammox and enhance the nitrogen removal efficiency of urban WWTPs. This process has been studied for treating urban wastewater with COD/N ratios ranging from 1.2 to 7.9. The proportion of N_2_O emissions (liquid and gaseous) from the anoxic zone to nitrogen loss is less than 0.08%. With the extension of reaction time, the proportion of N_2_O emissions to nitrogen loss further decreased to <0.02%, and the N_2_O conversion rate was significantly lower than the traditional denitrification process summarized in this article. This indicated that the addition of anammox could help reduce N_2_O emissions in BNR (Li et al., 2019).

## 5 New biological nitrogen removal process suitable for low C/N ratio wastewater to reduce N_2_O emissions

### 5.1 Anammox based processes

#### 5.1.1 PN/A or PD/A process

Based on Section 2.3, it can be seen that the average N_2_O emission level of anammox related processes in treating urban wastewater is lower than that of traditional BNR processes. However, the combination of processes and operating conditions also significantly influence the N_2_O conversion rate. In combined processes, such as PN/A and partial denitrification/anammox (PD/A), N_2_O accumulation is more significant. When the two-stage PN/A process was employed to treat high ammonium synthesis wastewater or low C/N mainstream wastewater, the comprehensive N_2_O conversion rate ranged from 4.1% to 6.6%, with most of the emissions being contributed by the PN unit (Desloover et al., 2011; Okabe et al., 2011; Li L et al., 2020). The comprehensive discharge of unipolar PN/A when treating urban wastewater accounted for 0.35%–2.00% of the influent nitrogen load, thereby enhancing the N_2_O emission reduction performance to some extent (Ali et al., 2016; Yang et al., 2016; Zhou et al., 2020).

Summarizing multiple studies, it has been observed that the PD/A process can achieve stable denitrification of mainstream wastewater with C/N ranging from 1.77 to 3.4 (Zhang et al., 2019). Comparing this process reveals that the maximum cumulative amount of N_2_O is 2.4% of influent nitrogen, which is lower than the traditional BNR or two-stage PN/A process (Du et al., 2020; Gao et al., 2022). The PN/A or PD/A combination process can help reduce N_2_O emissions during BNR of low C/N wastewater.

#### 5.1.2 Unipolar anammox

Although there are anammox, nitrifying bacteria and denitrification bacteria in the unipolar anammox system, the abundance of anammox is the highest, and the theoretical N_2_O emission value is low. The experiment showed that when treating wastewater with a C/N of 0.3∼1.4, the production of N_2_O was directly proportional to the filtration rate, with the N_2_O emission factor increasing from 0.012% at 1.0 m/h to 0.496% at 3.0 m/h. When the filtration rate was 1.5 m/h, both the removal rates of NH_4_^+^-N and NO_2_^−^-N reached 99%, and the N_2_O concentration was minimal. These conditions can be considered as the optimal reference for the process (Li et al., 2022).

#### 5.1.3 Feammox

Iron based materials enhancing BNR are considered one of the potential methods for effectively treating low C/N ratio wastewater. And feammox is a novel BNR process that combines anammox with Fe(III) reduction. Feammox can utilize Fe (III) instead of NO_2_^−^- N as the electron acceptor to reduce NH_4_^+^-N to N_2_, NO_2_^−^-N and NO_3_^−^-N through microorganisms (Peng et al., 2021). Therefore, the theoretical avoidance of N_2_O producing from denitrification is achieved. The feammox process has been demonstrated to be applicable for low C/N wastewater treatment. The feammox process operating in the Multistage Feammox Bioreactor (MSFB) can effectively treat actual anaerobic digestion (AD) wastewater. When the C/N ratio was 2.5, the AD effluent performance of the reactor exhibited the best, with a TN removal rate of 99% (Nguyen et al., 2023).

During the batch test of feammox, it was detected that the total N_2_O production was significantly lower (*p* < 0.05) than N_2_, and N_2_O emissions were beneath the detection limit, thereby confirming the potential to reduce N_2_O emissions (Zhou et al., 2016). However, in practical wastewater treatment, there remains a dearth of monitoring N_2_O emissions within the feammox system and the analysis of the impact of iron on N_2_O metabolic pathways.

#### 5.1.4 Denitrification anaerobic methane oxidation coupled with anammox process (DAMO-A)

In 2006, Islas-Lima S and Raghoebarsing found and obtained the concentration of n-DAMO for the first time. The n-DAMO group can be mainly divided into the DAMO archaea system of ANME-2d and the n-DAMO bacteria within the NC10 phylum. CH_4_ can serve as the carbon source to facilitate the denitrification process, convertingNO_3_^-^ to NO_2_^−^ and NO_2_^−^ to N_2_, respectively (Islas-Lima et al., 2004; Raghoebarsing et al., 2006). When co-cultured with anammox, a DAMO-A process can be formed. N-DAMO microorganisms are capable of oxidizing methane and releasing electrons during the reverse process of methane production. These electrons can be utilized as donors for denitrification, ultimately converting CH_4_ into CO_2_. Additionally, anammox will transform the generated NO_2_^−^ and NH_4_^+^ into N_2_ (Haroon et al., 2013). The reaction scheme of the system is as follows:

$${\text{CH}}_{4}+{\text{NO}}_{3}^{-}-N\to \;{\text{NO}}_{2}^{-}-N+H_{2}O,$$

$${\text{CH}}_{4}+{\text{NO}}_{2}^{-}-N\to \;N_{2}-N+H_{2}O,$$

$${\text{NH}}_{4}^{+}-N+{\text{NO}}_{2}^{-}-N\to \;N_{2}+{\text{NO}}_{3}^{-}-N.$$

The coupling process is an autotrophic system, and the functional microbial community n-DAMO and anammox mainly utilize inorganic carbon sources. Therefore, the low COD/N ratio of the influent should not affect the BNR performance, as it minimizes the likelihood of N_2_O generation through denitrification. Moreover, this process can simultaneously reduce CH_4_ and N_2_O emissions, offering a synergistic effects of pollution reduction and carbon reduction, making it a promising choice for future green innovations in WWTPs.

**FIGURE 5:**
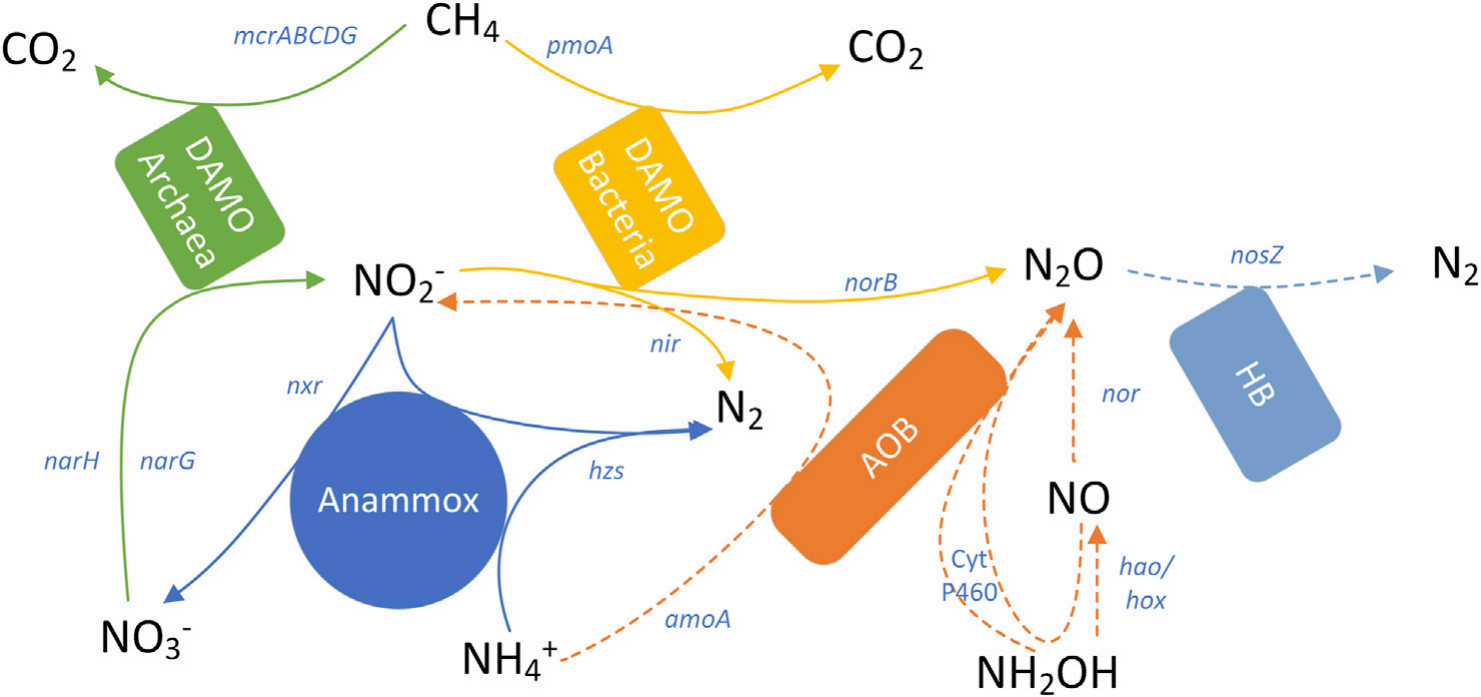
Carbon and nitrogen conversion process of DAMO-A. Solid line: a known process, dashed line: an unproven and possibly existing process.

However, limited systematic research exists on the emission and mechanism of N_2_O within the DAMO-A system. In 2015, it was reported that N_2_O was undetectable in the reactor on the 53rd, 115th, 199th, and 260th day (data not shown) (Hu et al., 2015). In 2019, researchers developed a new technology in MBfR that integrates PN, anammox, and methane dependent nitrite/nitrate reduction reactions, which can be abbreviated as PNAM process. The average TN removal rate achieved by this process was 98%, with a N_2_O emission factor of 0.34%, which is more likely related to AOB metabolism (Liu et al., 2019; Nie et al., 2020; Liu et al., 2021b). However, some scholars have pointed out that NC10 bacteria have a potential pathway to reduce NO to N_2_O, and the conversion rate of N_2_O is related to the external NO_2_^−^ concentration and the non-specific oxidation process of NH_4_^+^(Nie et al., 2019). Moreover, the genome of the DAMO-A system contains the gene of N_2_OR enzyme (Figure 5), but the specific role of this enzyme in the *in-situ* N_2_O conversion rate of the system has not been explored (Cogert et al., 2019). Therefore, in the future, it is still necessary to further quantify the gas emissions from this process in urban mainstream wastewater treatment, determine the N_2_O emission factors and its metabolic pathway of DAMO-A related BNR processes, and evaluate their comprehensive GHG emission reduction effects of eliminating CH_4_ and reducing N_2_O.

### 5.2 Enhanced process

#### 5.2.1 Sludge-derived hydrochar (SDHC)

Recycling Sludge-derived hydrochar (SDHC) enhances the denitrification effect of secondary effluent from WWTPs with low C/N and reduce N_2_O emissions. When C/N ranges from 3.0 to 3.2, the nitrogen removal rate (NRR) in the enhanced denitrification process DN-SDHC is 3.6 times higher than that of denitrification alone (DN). The high conductivity of SDHC accelerates the extracellular electron transfer from the carbon source to denitrification bacteria. SDHC also promotes a significant increase in the nosZ gene encoding N_2_OR, which is beneficial for reducing N_2_O accumulation (Hao et al., 2022).

#### 5.2.2 New strains used to strengthen BNR process

Aerobic environments are usually not conducive to reducing N_2_O emissions in WWTPs. Researchers have isolated a new strain *Pseudomonas* sp. YR02 that can reduce N_2_O under aerobic conditions. The successful amplification of four denitrification genes proved its complete denitrification ability. YR02 exhibited excellent performance in treating wastewater with high ammonia nitrogen and dissolved N_2_O. Although the strain achieves maximum inorganic nitrogen (IN) removal efficiency (>98%) under higher C/N conditions (C/N = 15), it was more conducive at reducing N_2_O emissions when the C/N ratio is 5. YR02 could reduce N_2_O emissions by 98.7% and increase NRE by 32% in WWTPs, demonstrating its potential for alleviating N_2_O emissions (Wang et al., 2023).

*Pseudomonas* sp. GZWN4, another species of the same genus, exhibits excellent aerobic denitrification performance across a wide range of C/N ratios for 5 to 20. This strain was isolated from seaweed aquaculture wastewater and is suitable for enhancing the treatment of ordinary or saline aquaculture water. GZWN4 carries the nosZ gene, indicating its ability to reduce N_2_O (Su et al., 2021). In addition, *Achromobacter* sp. HNDS-1 and *Enterobacter* sp. HNDS-6possess Nxr, narG, nirK, norB, and nosZ genes involved in the denitrification pathway, allowing them to effectively remove mixed nitrogen under C/N = 5 conditions. However, the N_2_O emission reduction effects of these three strains in actual sewage treatment have not been evaluated (Liu X et al., 2023).

For environments with low C/N ratios, *Bacillus thuringiensis* strain WXN-23, isolated from aquaculture filtrate, has stronger adaptability. Batch tests showed that the bacterium can achieve a TN removal rate of 95.996% at a C/N ratio of 5.91. Moreover, it has a relatively complete nitrification and denitrification pathway (NH_4_^+^-N→NH_2_OH→NO_2_^−^-N→NO_3_^−^-N→NO_2_^−^-N→NO→N_2_O→N_2_) (Xu et al., 2021), which promotes N_2_O reduction. Therefore, strain WXN-23 holds potential as a powerful strain for enhancing the green process in low C/N wastewater treatment.

Adding new bacterial strains to existing processes to enhance BNR performance has emerged as a research hotspot in recent years. Many new strains are isolated from environmental systems, sludge, or sewage treatment facilities and optimized for cultivation conditions. Before applying new bacterial strains, it is essential to assess their safety. There have been reports indicating that certain bacteria can produce hemolysin, which can cause toxic effects like cell membrane damage and lysis (Mogrovejo et al., 2020; Su et al., 2021). The presence of hemolysin production in a strain can serve as a crucial criterion for evaluating its safety.

However, if we want to apply this approach to enhance the process of sewage treatment plants and solve the problem of N_2_O emission reduction in low C/N wastewater, more in-depth experiments and research are still needed. For example, how to better maintain the strain in the system without loss with effluent. In actual sewage, there is a presence of diverse organisms, including predatory protozoa and phage, which can hinder the survival of introduced strains and potentially lead to the failure of bioaugmentation (Ma et al., 2022). To address this challenge, it is possible to select specific strains with BNR capabilities, such as the denitrifying strain *Alcaligenes aquatilis* AS1 (Cao et al., 2023). These strains can help enhance the interaction within microbial networks and promote the stability of microbial communities. Another approach to ensure the survival of selected strains involves immobilizing them using gel particles or other immobilized redox mediator granules (IRMG), which prevents competition-induced destruction. The application of this biological immobilization technology can enhance the survival capacity of the strains (Han et al., 2021; Shi et al., 2022; Sun et al., 2023). It is also important that the majority of aerobic denitrification strains thrive in environments with an ample carbon source. Therefore, when evaluating the trade-off between NRE and the reduction of N_2_O emissions in low C/N environments, this factor must be taken into account.

## 6 Conclusion

Based on the characteristics of biological nitrogen removal (BNR) processes, such as complete nitrification/denitrification, partial nitrification/denitrification, and anammox, it has been observed that treating domestic wastewater with a generally low C/N ratio leads to higher N_2_O emissions. The insufficient carbon sources and a low carbon loading rate in low C/N wastewater intensify the competition among denitrification enzymes, affecting the production and consumption of N_2_O and resulting in its accumulation. Additionally, the limitation of inorganic carbon (IC) in autotrophic nitrification/denitrification systems restricts the oxidation activity of ammonia-oxidizing bacteria (AOB) towards NH_4_^+^ and contributes to increased N_2_O emissions. To effectively reduce N_2_O emissions in low C/N wastewater, adjusting the type or dosage of the carbon source has proven to be effective. Furthermore, during the upgrade process of sewage treatment plants, comprehensive optimization of aeration methods, internal circulation ratio, and other conditions, as well as the implementation of oxidation ditch systems and biofilm systems, can be implemented to reduce N_2_O emissions. The development of new BNR processes, particularly autotrophic anammox-related processes such as unipolar PN/A, PD/A, unipolar Anammox, and Feammox, shows promise in reducing the demand for organic carbon and achieving deep denitrification and N_2_O emission reduction in low C/N wastewater. Notably, the DAMO-A system can utilize CH_4_ while reducing N_2_O production. However, there is still a lack of systematic research on the emission factors and metabolic mechanisms of N_2_O in these processes, which would provide theoretical support for emission reduction.

## 7 Future outlook

The perspective of this review focuses on N_2_O emission reduction through BNR of low C/N wastewater. We have proposed some directions and suggestions to enhance our understanding and develop solutions for mitigating N_2_O emissions in low C/N wastewater:

• Develop N_2_O emission evaluation indicators for WWTPs or BNR processes. This includes measuring the N_2_O conversion rate of the process and establishing standardized measurement methods for different nitrogen-based unit conversion rates. Additionally scientific methods should be established to compare the effectiveness of N_2_O emission reduction strategies across different processes.
• Explore the application of emerging methodologies such as SCRS in studying microbial N_2_O metabolism. This approach can help analyze the N_2_O production and reduction functions of individual functional microbial communities in complex microbial systems involved in BNR. It is advisable to consider multiple factors such as microbial community structure, N_2_O metabolic characteristics, the relationship between electricity competition caused by carbon source limitation and enzyme activity. More in-depth consideration of various N_2_O production pathways and the contributions of representative microorganisms should be formed to address the limitations of N_2_O metabolism research in low C/N wastewater treatment.
• When proposing N_2_O emission reduction strategies, more consideration should be given to the economic and cost-effectiveness of measures such as adding carbon sources and changing feeding methods. These measures should not only provide environmental benefits but also be evaluated in terms of emission reduction scenarios, such as carbon footprint. Conducting a comprehensive review of emission reduction plans will facilitate their practical application.
• Further investigation into novel processes, such as the unipolar and two-stage systems driven by Anammox, is warranted. Of particular interest is the DAMO-A process, which has the potential to simultaneously reduce CH_4_ and N_2_O emissions. This process mainly focuses on autotrophic DAMO microorganisms and Anammox bacteria. In the future, rapid enrichment methods need to be developed to explore their N_2_O emission levels and mechanisms as application support.

In summary, the scientific issues addressed in this article are of great significance in the context of global warming. As an essential aspect of sustainable social development, sewage treatment must strive to achieve the objective of coordinated pollution reduction and carbon reduction. For urban wastewater that has large displacement, we need to pay attention to the powerful influencing factors of its low C/N ratio, and further analyze the impact and mechanism of this characteristic on N_2_O emissions in the BNR process. Research should not be limited solely to monitoring emissions on a macro-scale. In order to address this challenging issue, it is necessary to develop more robust functional analysis methods, surpass the limitations posed by the complexity of environmental microbial systems and various BNR processes, and expand future research in this direction.
